# Proof-of-concept study of clinical use of blood-based MAVS biosensor in predicting immunotherapy response in SCLC patients

**DOI:** 10.1016/j.tranon.2026.102756

**Published:** 2026-04-04

**Authors:** Luisa Amato, Ines Tavoletta, Caterina De Rosa, Mimimorena Seggio, Francesco Arcadio, Sara Capaldo, Faiz Ul Haq, Concetta Tuccillo, Carla Esposito, Gaetano di Guida, Francesca Iommelli, Viviana De Rosa, Alfonso Reginelli, Salvatore Cappabianca, Floriana Morgillo, Fortunato Ciardiello, Valerio Nardone, Nunzio Cennamo, Carminia Maria Della Corte, Luigi Zeni

**Affiliations:** a Department of Precision Medicine, University of Campania Luigi Vanvitelli, 80131 Naples, Italy; b Department of Engineering, University of Campania Luigi Vanvitelli, 81031 Aversa, Italy; c Department of Engineering, Telematic University Pegaso, 80132 Naples, Italy; d Institute of Biostructures and Bioimaging, National Research Council, 80145 Naples, Italy; e Radiology and Radiotherapy, Department of Precision Medicine, University of Campania Luigi Vanvitelli, 80138 Naples, Italy

**Keywords:** SCLC, Blood-based biomarker, Liquid biopsy, MAVS, Immune sensing

## Abstract

Small cell lung cancer (SCLC) is an aggressive neuroendocrine tumor with poor prognosis and limited benefit from immune checkpoint inhibitors (ICIs). Biomarker-driven patient stratification has been hindered by small biopsy samples, high tumor heterogeneity, and the limited predictive value of PD-L1 and tumor mutation burden. Mitochondrial antiviral-signaling protein (MAVS) has emerged as a potential immune activation marker, particularly in patients receiving DNA-damaging therapies. We report a proof-of-concept clinical study evaluating a surface plasmon resonance–plastic optical fiber (SPR‑POF) biosensor functionalized with anti-MAVS antibodies to detect the protein in serum from SCLC patients undergoing chemo-immunotherapy, with or without radiotherapy. The biosensor achieved a limit of detection of 0.13 nM in human diluted serum and demonstrated high selectivity against common serum proteins. In a cohort stratified as best responders (disease control >6 months) and non-responders (progressive disease as best response), MAVS levels measured in responders were on average tenfold higher than in non-responders, consistent with previous preclinical PBMC and western blot data. The SPR‑POF platform demonstrated portability, cost-effectiveness (estimated 5 USD/unit), and operational simplicity, highlighting its potential for point-of-care testing (POCT) applications. Although limited by small patient numbers, these findings support MAVS as a promising predictive biomarker in SCLC, warranting validation in larger prospective studies.

## Highlights

• SPR‑POF biosensor detects serum MAVS in SCLC chemo-immunotherapy patients.
• MAVS levels 10 × higher in best responders vs non-responders SCLC patients.
• Biosensor LOD 0.13 nM with high selectivity.
• Portable, low-cost platform suitable for POCT.
• First clinical application of MAVS biosensing in oncology.

## Graphical abstract

**Figure:**
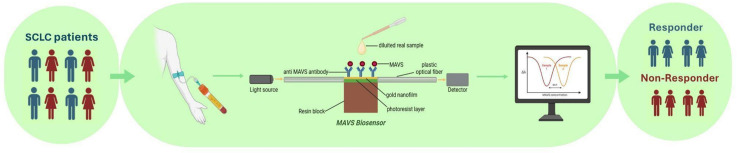

## Introduction

Small cell lung cancer (SCLC) is a highly aggressive and heterogeneous neuroendocrine malignancy characterized by rapid growth, early metastasis, and poor prognosis, with a 5-year overall survival rate of approximately 7 % [1]. Despite initial sensitivity to chemotherapy, most patients experience disease progression due to acquired resistance [2]. The advent of immunotherapy, particularly immune checkpoint inhibitors (ICIs), has provided some improvement in survival; however, the benefit is limited to a small subset of patients, and the duration of response remains brief [3]. This underscores the urgent need for novel biomarkers that can accurately predict and monitor therapeutic responses, enabling early identification of responders and non-responders to chemoimmunotherapy in extensive-stage SCLC.

Previous work has shown that treatment with DNA-damaging therapies in peripheral blood mononuclear cells (PBMCs) from SCLC patients undergoing chemoimmunotherapy enhances the interaction between Stimulator of Interferon Genes (STING) and Mitochondrial Antiviral Signaling protein (MAVS), suggesting a potential biomarker role for this complex [4]. While STING has been more extensively studied in cancer immunity [[5], [6], [7]], the role of MAVS as a surrogate biomarker for immune response in SCLC remains underexplored. Some evidence indicates that MAVS expression is upregulated in the peripheral blood of patients who exhibit prolonged response to therapy, implying its potential as a dynamic biomarker reflective of immune activation [8].

In this context, the development of point-of-care testing (POCT) strategies emerges as a crucial step in translating biomarker discovery into clinical practice. Among them, surface plasmon resonance (SPR) biosensors, particularly those combined with plastic optical fibers (POFs) technology, enable rapid, label-free and highly sensitive detection of circulating proteins directly from small volumes of serum [9].

SPR-based technologies have already been applied for circulating cancer biomarkers, including circulating tumour DNA (ctDNA), microRNAs (miRNAs), circulating tumour cells (CTCs), lipids, and proteins [10,11]. This makes SPR a promising candidate for diagnostics based on liquid biopsies. Moreover, recent advancements in SPR sensor design indicate that portable, miniature platforms for real-time biomolecular detection are achievable, facilitating potential clinical applications [12]. Plasmonic optical fiber biosensors exploiting Tilted Fiber Bragg Grating (TFBG) technology have effectively been employed in the detection of lung cancer biomarkers, such as HER2, highlighting the applicability of fiber-based SPR sensing in respiratory oncology [13]. Additionally, advanced developments are realizing miniaturized, smartphone-compatible SPR devices that enable rapid, decentralized testing in resource-limited or point-of-care scenarios [14]. Technological advancements facilitate the development of customized SPR-POCT platforms for monitoring novel immune system-related biomarkers, including MAVS, in SCLC.

In this work, we present the development of a sensitive SPR-POF biosensor specifically designed to detect MAVS in the serum of SCLC patients undergoing chemo-immunotherapy. By comparing MAVS levels between responders and non-responders, we aim to assess its potential as a predictive biomarker. This approach combines the clinical need for accurate, non-invasive monitoring of tumor-immune dynamics with the feasibility of cost-effective POCT technologies.

## Materials and methods

### Chemicals

N—Hydroxysuccinimide (NHS), N-(3-Dimethylaminopropyl)-N′-ethylcarbodiimide hydrochloride (EDC), (±)-α-Lipoic acid, ethanolamine, phosphate buffered saline (PBS) were purchased from Merck KGaA (Darmstadt, Germany); Anti-MAVS antibody (D5A9E) was purchased from Cell Signaling technology (Danvers, MA, USA); Recombinant Human Mavs Protein (ab139779) was purchased from ABCAM (Cambridge, CB2 0AX, UK).

### SPR-POF platform realization

The SPR-POF platform manufacturing process is previously described [15]. Briefly, a POF was embedded in a resin support and polished with polishing papers with different grits (5 μm and 1 μm grits) to realize the d-shaped region (length=1 cm). Subsequently, a high refractive index (RI) photoresist buffer layer (Microposit S1813, MicroChem Corp., Westborough, MA, USA), of about 1 μm thick, was spin coated to improve the optical performance [15]. Finally, a 60 nm gold film was deposited by a sputter coater machine (Safematic CCU-010, Zizers, Switzerland).

**Fig. 1:**
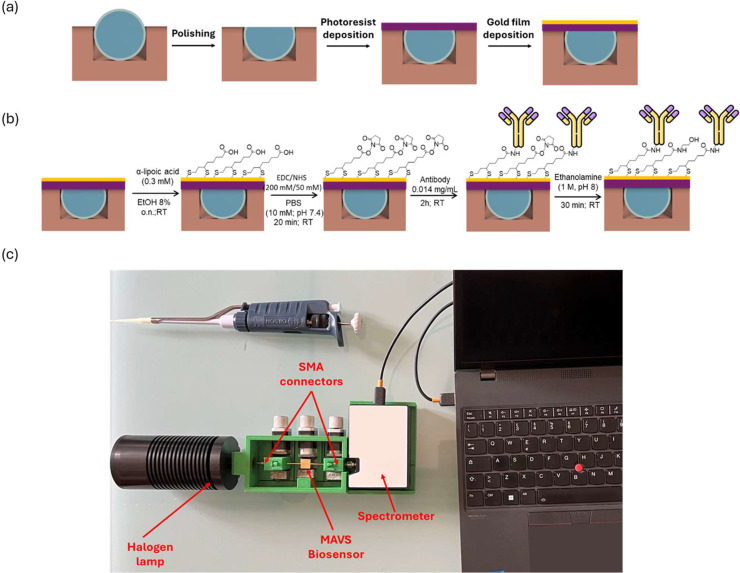
a) Scheme of the SPR-POF probe fabrication procedure; b) Scheme of the SPR-POF functionalization process with anti-MAVS antibody; c) Experimental setup used to test the MAVS biosensor.

A schematic view of the SPR-POF sensor manufacturing process is shown in Fig. 1a.

### Functionalization of the plasmonic surface

The SPR surface functionalization was carried out according to the procedure previously reported [16]. At first, the gold surface was extensively rinsed with Milli-Q water. After that, the gold nanofilm was treated overnight (o.n.) at room temperature (RT) with α-lipoic acid (0.3 mM in 8 % ethanolic solution) to achieve an α-lipoic acid-based self-assembled monolayer (SAM). Then, the α-lipoic acid carboxylic groups were activated with EDC/NHS (200 mM/50 mM, respectively) in PBS, pH 7.4, for 20 min at RT. After washing three times with PBS to remove excess reactant, the surface was incubated for 2 h at RT with anti-MAVS antibody (0.014 mg/mL). Finally, the surface passivation was performed by incubating ethanolamine (1 M, pH 8.0) for 30 min at RT, in order to quench unreacted activated carboxylic groups. The functionalized platforms were washed in PBS and stored in PBS at 4 °C. The scheme of platform functionalization process is reported in Fig. 1b

### Experimental setup

To monitor the biosensor response, a versatile and low-cost experimental setup was used. More specifically, a halogen lamp (HL-2000-LL, Ocean Optics, Orlando, FL, USA) with an emission range of 360–1700 nm was employed as white light source. A spectrometer (SR-6VN500, Ocean Optics, Orlando, FL, USA) with a detection range 300–1000 nm was used as a detector. The MAVS biosensor was connected to the light source and the spectrometer through SMA connectors. Moreover, the realized biosensor was immobilized in a 3D-printed holder. Fig. 1c provides an image which details the experimental setup described.

### Patient enrollment and grouping

Patients diagnosed with extensive‑stage SCLC who were receiving chemotherapy (cisplatin or carboplatin, etoposide) and/or anti‑PD‑L1 antibody therapy (atezolizumab, durvalumab), with or without radiotherapy, were enrolled in this study (*n* = 8). Radiotherapy was administered either with palliative intent to pleural or brain lesions in non‑responders, or as stereotactic treatment to pulmonary nodules in best responders. Patients were stratified into two groups: best responders (BR), defined as those with disease control lasting longer than 6 months, and non‑responders (NR), defined as patients exhibiting progressive disease (PD) as their best response to treatment. Detailed patient characteristics are reported in Table 1.

### Ethics statement and sample collection

All human samples were collected after obtaining written informed consent from the patients, in compliance with the Declaration of Helsinki. The study protocol was reviewed and approved by the Ethics Committee of the University of Campania “Luigi Vanvitelli,” Naples (approval No.6691 in March 2025). Peripheral blood samples were collected from patients in BD vacutainer tubes spray-coated with K2EDTA (BD, Franklin Lakes, NJ, USA). Blood serum was obtained by centrifugation, aliquoted, and immediately stored at −20 °C for subsequent MAVS quantification.

### Cell line and MAVS small RNA interference (siRNA)

The SCLC cell line NCI-H1688 (ATCC CAT#CCL-257) were maintained in RPMI 1640 supplemented with 10 % FBS and 1X penicillin–streptomycin in a humidity-controlled environment (37 °C, 5 % CO^2^). Cell lines were obtained from and authenticated by the American Type Culture Collection (ATCC). Cell lines morphology was monitored, and the cell lines were routinely tested for mycoplasma using a mycoplasma detection kit (InvivoGen).

For silencing experiments, MAVS-targeted siRNA (Silencer®, Thermo Fisher) and control non-targeting siRNA (Dharmacon Inc.) were used according to the manufacturer’s instructions. Briefly, cell suspension was plated at 40 % confluence and allowed to grow in a humidified incubator in 5 % CO^2^ at 37 °C for 24 h. Cells were then transfected with 100 nmol/L siRNAs using Dharmafect reagent (Dharmacon). After 72 h, cells were recovered and then lysed for western blot analysis.

### Western blot

Whole protein lysates from SCLC cells were obtained as previously described [17]. Briefly, homogenization was carried out in RIPA lysis buffer composed of 0.1 % sodium dodecyl sulfate (SDS), 0.5 % deoxycholate, 1 % Nonidet P-40, 100 mmol/L NaCl, 10 mmol/L Tris–HCl (pH 7.4), 0.5 mmol/L dithiothreitol (DTT), and 0.5 % phenylmethylsulfonyl fluoride (PMSF). This buffer was supplemented with a protease inhibitor cocktail (Hoffmann-La Roche, Basel, Switzerland) and phosphatase inhibitor tablets (PhosSTOP; Roche Diagnostics, Basel, Switzerland) to prevent protein degradation and dephosphorylation. Lysates were clarified by centrifugation at 2348 rcf for 20 min at 4 °C. Protein concentrations were normalized across samples. Equal amounts of whole-cell lysates were mixed with LDS reducing sample buffer (Thermo Fisher or equivalent), boiled at 100 °C for 10 min, and loaded onto SDS-PAGE gels for electrophoretic separation. Proteins were subsequently electrotransferred onto 0.2 µm nitrocellulose membranes (Trans-Blot Turbo Transfer System; BioRad). Membranes were blocked for 90 min at room temperature with a blocking buffer (e.g., 5 % non-fat dry milk or BSA in TBS-T). Primary antibody incubations were performed overnight at 4 °C using the following antibodies: anti-MAVS (D5A9E) (1:1000 dilution, Cell Signaling Technology cat#24,930), anti-GAPDH (D16H11) (1:1000, Cell Signaling Technology cat#5174). After washing, membranes were incubated with horseradish peroxidase (HRP)-conjugated secondary antibodies (anti-rabbit or anti-mouse IgG; BioRad) for 1 hour at room temperature. Protein detection was carried out using Clarity Western ECL Substrate (BioRad) and visualized with the ChemiDoc imaging system (BioRad). Band intensities were quantified using BioRad Image Lab software version 3.0.1. Original uncropped gels are reported in the Supplementary Material file.

### MAVS biosensor measurement protocol and data treatment

The measurement protocol employed for testing the MAVS biosensor was both rapid and straightforward, requiring small volumes of solution (approximately 50 µL). Specifically, the biosensor response was monitored at increasing MAVS concentrations (ranging from 0.25 nM to 500 nM) diluted in PBS or in real matrix (serum from a healthy patient diluted in PBS 1:500). Each MAVS solution was dropped onto the sensitive surface of the biosensor and left to incubate for 10 min at RT to ensure interaction between the target analyte (MAVS protein) and the bioreceptor (anti-MAVS antibody). Then, washing steps with PBS were performed to remove any non-specific binding, and the transmitted spectra were recorded with PBS (blank) as a bulk. More in detail, experimental data were acquired using proprietary software (OceanView, version 2.0.16, Ocean Optics, Orlando, FL, USA) and then processed with MATLAB software (version R2022b, MathWorks, Natick, MA, USA). In particular, the SPR spectra were achieved by normalizing the transmitted spectra (acquired in PBS as bulk) to the one acquired in air (reference spectrum), where the SPR condition is not satisfied [15].

Variations in the resonance wavelength compared to that of the blank solution (i.e., PBS or human diluted serum without MAVS), for each tested MAVS concentration, were used to achieve the dose-response curves (in PBS and in human diluted serum).

Specifically, the dose-response curves were obtained by fitting the experimental values with the Langmuir equation:

(1) $$\Delta \lambda_{c}=\lambda_{c}-\lambda_{0}=\Delta \lambda_{max}\cdot \left(\frac{c}{c\;+\;K}\right)$$

where c is the MAVS concentration, $\lambda_{c}$ is the recorded resonance wavelength for a standard solution at concentration c, $\lambda_{0}\;$is the signal for a solution at zero concentration of MAVS (blank solution), $\Delta \lambda_{max}$ is the maximum value of $\Delta \lambda_{c}$ obtained at the saturation value, and the parameter K is the reciprocal of the affinity constant $\left(K_{aff}\right)$ of the bioreceptor for the target analyte. All the Langmuir fitting parameters were obtained by using OriginPro 9 software (Origin Lab. Corp., Northampton, MA, USA).

Moreover, at low concentrations, when c is much smaller than K, Eq. (1) can be approximated as a linear model and the slope ($\Delta \lambda_{max}$/ K) is defined as the sensitivity at low concentrations ($S_{low-conc}$). From the linear model at low concentrations, the limit of detection (LOD) can be estimated as the ratio between the standard deviation of the response at the blank solutions $(\lambda_{0})\;$multiplied by 3.3 and the $S_{low-conc}$ [18].

The two control samples (negative control and positive control verified via western blot), and ten real samples (serum from non-responder patients, named NR and serum from best responder patients, named BR) were tested with different dilution ratios to verify the absence or presence of MAVS protein. In particular, the negative sample was tested at different dilution ratios (1:5000, 1:1000, 1:500), and then, the positive sample was also tested (1:10,000, 1:5000, 1:2500, 1:1000, 1:500, and 1:250) of the same biosensor. The NR samples were tested at dilution ratios of 1:10,000, 1:5000, 1:1000, 1:500, 1:250, 1:100, and 1:50. The BR samples, potentially presenting higher concentrations of MAVS protein, were diluted at dilution ratios of 1:25,000, 1:10,000, 1:5000, 1:2500, 1:1000, 1:500 and 1:250.

## Results

### Patients characteristics

A total of *n* = 10 samples from eight patients diagnosed with SCLC were included in the analysis, representing opposite clinical outcomes (NR and BR, respectively). NR patients with PFS <6 months were *n* = 5, while BR patients with PFS>6 months were *n* = 5, with 1 patient included with PFS >6 years (identified as long immune responder, LIR) (Table 1). The study cohort comprised a total of eight patients (*n* = 7 males and *n* = 1 female) with a median age of 63 years (range: 50–68). All patients were diagnosed with advanced disease, specifically *n* = 7 with SCLC-ES and *n* = 1 with small cell neuroendocrine carcinoma. The therapeutic landscape for the cohort was characterized by first-line chemoimmunotherapy (using either Durvalumab or Atezolizumab in combination with Cisplatin or Carboplatin and Etoposide). The median progression-free survival (PFS) for first-line treatment was 7.5 months (range: 5–11 months, excluding the long-term responder). Following first-line progression, 75 % of the cohort (6/8 patients) required subsequent lines of therapy, including Lurbinectedin, Gemcitabine, Topotecan, or Irinotecan. Notably, the cohort included *n* = 1 Long Immuno-Responder (LIR), a 68-year-old male who has achieved an exceptional response duration of over 6 years on Atezolizumab maintenance therapy. Radiotherapy (RT) was a frequent component of clinical management, administered to 75 % of the patients (6/8). These treatments included consolidative thoracic RT (66 Gy), whole-brain RT (30 Gy) for metastases, and stereotactic RT for oligoprogression in the lungs and adrenal glands. Longitudinal monitoring was supported by blood collection at key clinical milestones, specifically at points of Response and Disease Progression (PD), to facilitate molecular analysis.

**Table 1:** Patients’ demographic and clinical characteristics.

Histology	Age	Sex	Diagnosis Date	Therapy	Radiotherapy: Date and Details	PFS	Sample name
Small cell neuroendocrine carcinoma	66	M	03 Aug 2023	1st line: Cisplatin + Etoposide + Durvalumab. 2nd line: Lurbinectedin; 3rd line: Gemcitabine	Radiotherapy 9 Gy total in 3 fractions (300 cGy/day), VMAT 6 MV, lesion in pleura/costovertebral recess (right), interrupted early (planned 30 Gy/10 fractions); Radiotherapy, 27 Gy total in 3 fractions (900 cGy/day), VMAT-SRT 6 MV, lesion in left parietal brain region	1st line: 9 months. 2nd line: 3 months	NR#1
Small cell lung carcinoma, extensive stage	55	M	27 Jul 2023	1st line: Carboplatin + Etoposide + Atezolizumab (5 cycles); Maintenance: Atezolizumab every 21 days	Radiotherapy: 60 Gy total dose, stereotactic technique, target: nodules in apical segment of right upper lobe (SUV max 6)	1st line: 6 months	BR#1
Small cell lung carcinoma, extensive stage (SCLC-ES)	62	M	11 Sep 2023	1st line: Carboplatin + Etoposide + Atezolizumab. 2nd line: Gemcitabine.	None Reported	1st line: 5 months; 2nd line: 3 months.	NR#2
Small cell lung carcinoma, extensive stage (SCLC-ES)	68	M	10 Dec 2018	1st line: Carboplatin + Etoposide + Atezolizumab. Maintenance: Atezolizumab (10 cycles). Rechallenge 1st line. 2nd line: Ifinatamab deruxtecan.	RT to the anterior segment of the LUL (Left Upper Lobe). RT to a nodule in the RUL (Right Upper Lobe) for PD (Progressive Disease).	1st line: 10 months; Rechallenge: 7 months;	BR#2
Small cell lung carcinoma, extensive stage (SCLC-ES)	68	M	02 Jan 2024	1st line: Carboplatin + Etoposide + Durvalumab. 2nd line: Lurbinectedin (1 cycle).	None Reported	1st line: 6 months	NR#3
Small cell lung carcinoma, extensive stage (SCLC-ES)	68	M	10 Dec 2018	1st line: Carboplatin + Etoposide; 2nd line Carboplatin + Etoposide + Atezolizumab (ongoing Atezolizumab maintenance).	Radiotherapy concurrently with maintenance Atezolizumab	1st line: 10 months; 2nd line: >6 years	BR#3
Small cell lung carcinoma, extensive stage (SCLC-ES)	50	M	20 May 2024	1st line: Carboplatin + Etoposide + Durvalumab; 2nd line: Topotecan.	Radiotherapy to the primary lung lesion in the RUL (Right Upper Lobe), 25 fractions.	1st line: 7 months 2nd line: 3 months	BR#4; NR#4
Small cell lung carcinoma, extensive stage (SCLC-ES)	56	F	01 Mar 2023	1st line: Carboplatin + Etoposide + Durvalumab. 2nd line: irinotecan. 3rd line: Lurbinectedin.	Consolidative RT to the left lung and mediastinum, 66 Gy. Whole-brain RT (WBRT), 30 Gy. RT to the right adrenal gland, 20 Gy.	1st line: 11 months. 2nd line: 7 months	BR#5; NR#5

### Functionalization validation

To develop the MAVS biosensor a SPR-POF probe was functionalized with an anti-MAVS antibody, as extensively described in Section 2.3. The confirmation of the effective functionalization was assessed by evaluating the plasmonic spectral changes before and after the functionalization process, considering the same RI surrounding medium (i.e., PBS).

As illustrated in Figure S1 of the supplementary materials file, the SPR wavelength shows a clear shift towards higher wavelength values (red-shift). This behavior indicates an increase in the RI at the interface between the gold nanofilm and the dielectric medium (PBS) due to the immobilization of the antibody SAM over the SPR surface, compared to the non-functionalized surface, hence confirming the success of the immobilization procedure.

### Binding test in PBS and in diluted human serum

To evaluate the biosensor performance in MAVS detection, the proposed biosensor was tested at increasing concentrations of MAVS protein diluted in PBS, ranging from 0.25 to 500 nM, following the measurement protocol described in Section 2.9.

**Fig. 2:**
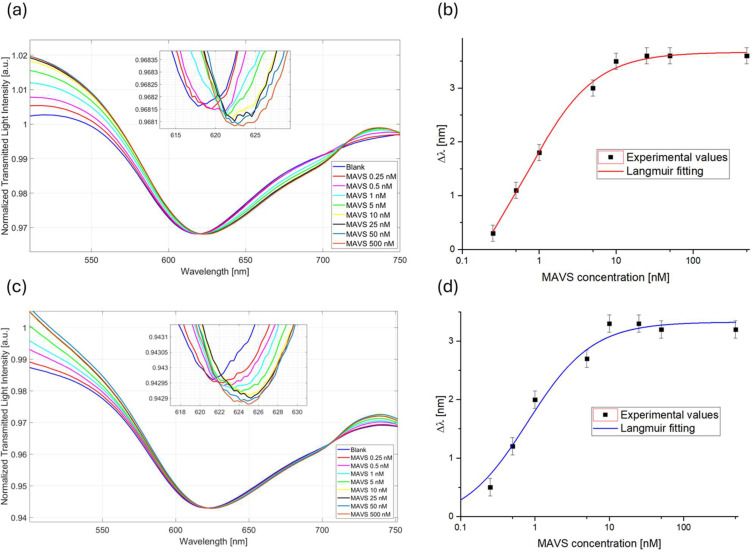
Binding tests. SPR spectra obtained in a) PBS and c) human serum diluted 1:500 at increasing MAVS concentrations**;** Dose–response curves obtained through MAVS monitoring in b) PBS and d) human serum diluted 1:500. The error bar (0.2 nm) corresponds to the maximum standard deviation obtained by testing five biosensors.

Specifically, Fig. 2a reports the SPR spectra obtained at different MAVS concentrations in PBS. In particular, as shown in Fig. 2a, the antibody-MAVS binding causes a red-shift in the resonance wavelength, symptoms of an increase in the RI of the receptor layer upon the gold surface in a similar way to a previous study [16].

The sensor saturation was reached at MAVS concentration of about 10 nM, as shown in Fig. 2a and 2b The experimental data, reported as the resonance wavelength variation (∆λ) at varying MAVS concentrations, were calculated with respect to the value achieved in PBS (in the absence of MAVS) and fitted with the Langmuir model equation (Eq. (1)). The achieved dose-response curve in PBS is reported in Fig. 2b Moreover, the error bars (about 0.2 nm), calculated as the maximum standard deviation obtained by testing five similar biosensors in similar working conditions, are also reported in Fig. 2b

To monitor the presence of MAVS in a real matrix, the developed biosensor was also tested in human serum. The serum used for the binding test in the real matrix was from a healthy patient (not treated with therapy) diluted with PBS 1:500. The same concentration range (from 0.25 to 500 nM) was explored, in accordance with the measuring protocol reported in Section 2.9. Also in this case, the antibody-MAVS binding causes a red-shift in the resonance wavelength, reaching the biosensor saturation at MAVS concentration of 10 nM, as shown in Fig. 2c. The corresponding dose-response curve in diluted human serum is shown in Fig. 2d Again, the experimental data are reported as resonance wavelength variation (∆λ) at varying MAVS concentrations, calculated with respect to the value achieved in diluted human serum (in the absence of MAVS), and fitted by Eq. (1). Langmuir fitting parameters achieved in both PBS and diluted human serum are reported in Table 2.

**Table 2:** Langmuir fitting parameters obtained for MAVS monitoring in PBS and diluted human serum.

Matrix	$\lambda_{0}\;\left[\mathrm{nm}\right]$	$\Delta \lambda_{\max}\;\left[\mathrm{nm}\right]$	K[nM]	Statistics	
				χ^2^	R^2^
PBS	−0.89±0.28	3.67±0.05	0.68±0.11	0.37	0.99
Diluted human serum	−0.09±0.17	3.33±0.09	0.79±0.16	1.46	0.98

The fitting parameters listed in Table 1 were used to calculate the biosensor’s analytical parameters for MAVS monitoring in both the tested matrices. Table 3 reports the results in terms of$\;S_{low-conc}$, LOD, and $K_{aff}$, calculated as described in Section 2.9.

**Table 3:** Analytical parameters of the biosensor obtained for MAVS detection in PBS and diluted human serum.

Matrix	LOD [nM]	$\;S_{\mathrm{low}-\mathrm{conc}}$ [nm/nM]	$K_{\mathrm{aff}}$ [nM]^−1^
PBS	0.17	5.39	1.47
Diluted human serum	0.13	4.22	1.27

From the comparison shown in Table 3, it is possible to confirm the almost absence of matrix effect in the measurement condition, since the biosensor performances obtained in the two matrices are very similar.

### Selectivity tests

**Fig. 3:**
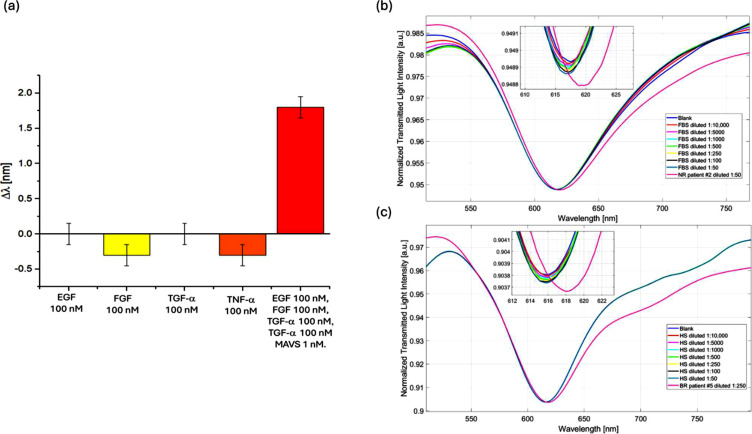
Selectivity tests. (a) Resonance wavelength variation (Δλ) obtained by testing the MAVS biosensor with four interfering human proteins (EGF, FGF, TGF-α, and TNF-α) (100 nM in PBS) and with a mixture of them (in PBS) containing also the target analyte (MAVS 1 nM). (b) SPR spectra recorded in FBS at various dilutions (1:10,000 to 1:50) and in a clinical sample from a NR patient (Case #2) diluted 1:50. (c) SPR spectra recorded in HS at various dilutions (from 1:10,000 to 1:50) and in a clinical sample from a BR patient (Case #5) diluted 1:250.

To verify and demonstrate the selectivity of the developed biosensor, four interfering proteins commonly found in human serum (EGF, FGF, TGF-α, and TNF-α) were tested at a concentration ten times higher than the saturation value of the biosensor (100 nM). As reported in Fig. 3a, the presence of interfering proteins shows no significant signal from the biosensor. Finally, a mixture of these interfering proteins (at a concentration of 100 nM each), containing MAVS at a concentration two orders of magnitude lower (1 nM), was also tested. In this case, as shown in Fig. 3, the presence of the target analyte (MAVS) results in a significant variation in resonance wavelength of approximately 1.8 nm, consistent with the dose-response curve in PBS. Furthermore, in order to assess the absence of complex matrix interferences, additional tests were carried out with fetal bovine serum (FBS) and human serum (HS) control. More specifically, both FBS and HS were diluted at the same dilution factors of NR patients to contemplate the worst-case scenario in terms of matrix complexity (due to lower dilution ratios used). As shown in Fig. 3b and Fig. 3c, no spectral shifts were achieved with FBS and HS, respectively, at different dilution factors. Oppositely, the biosensor’s response to real samples, namely NR patient (case #2) diluted 1:50 and BR patient (case #5) diluted 1:250, produced clear resonance wavelength shifts, as for SPR spectra reported in Fig. 3b and 3c, respectively. These results confirm the biosensor's ability to detect only the target analyte (MAVS) within complex human serum matrices across different clinical response profiles.

**Fig. 4:**
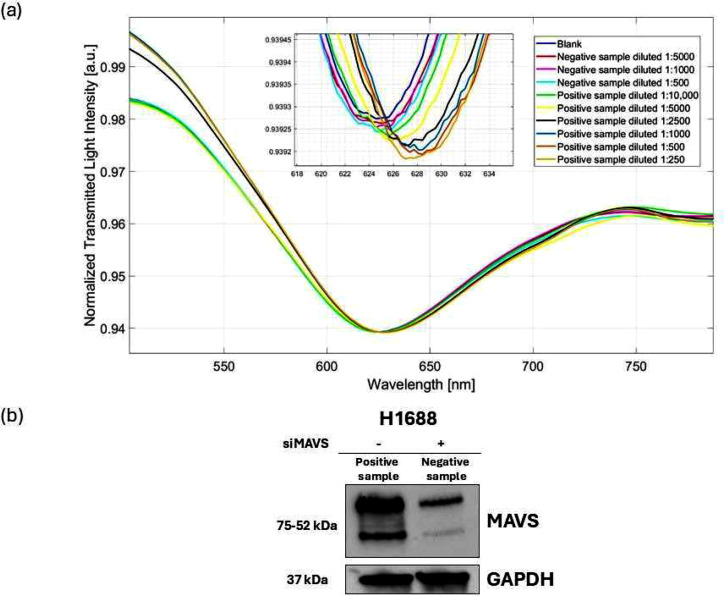
a) SPR spectra recorded in the presence of negative control first, and then the positive control, diluted with different dilution factors. b) Western blotting of total protein lysates for H1688 SCLC cell line with and without MAVS silencing. GAPDH was used to ensure equal loading. Original uncropped gels are reported in Figure S2.

Moreover, to confirm the selectivity of the proposed biosensor systems, the MAVS biosensor was tested with control samples following the measurement protocol described in Section 2.9 and also analyzed via western blot. Specifically, as shown in Fig. 4a, the presence of the negative sample did not produce any response from the biosensor. On the other hand, the positive sample registered a significant signal from a dilution of 1:10,000, due to the presence of MAVS, up to saturation (positive sample diluted 1:1000), in line with the results of the western blot (Fig. 4b).

### MAVS detection in real samples

**Fig. 5:**
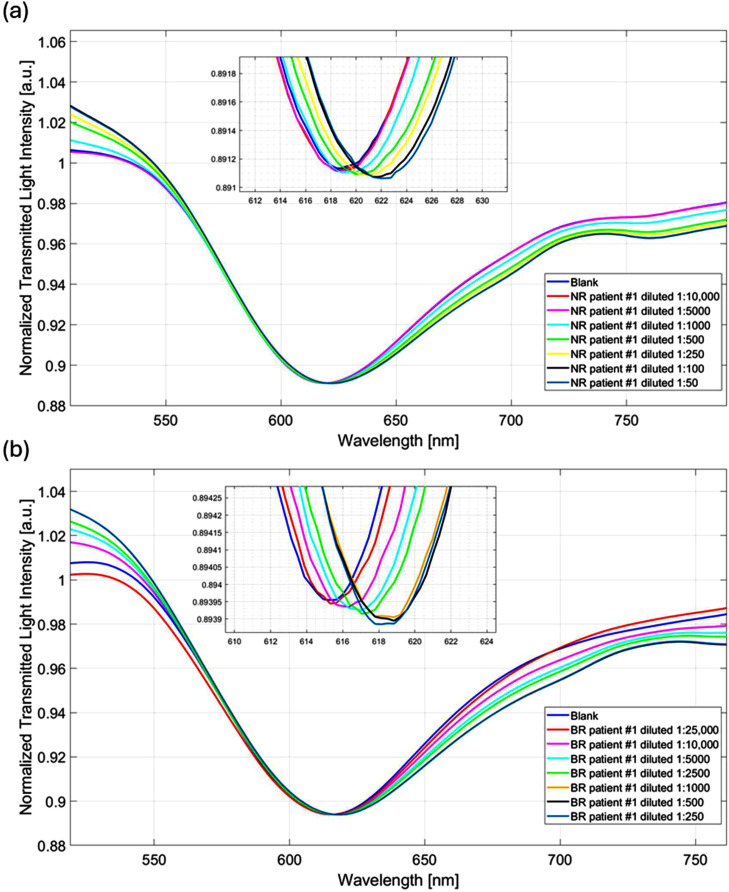
a) SPR spectra recorded in the presence of the different dilution ratios of a) the NR patient #1 and b) the BR patient #1.

The MAVS biosensor was also used to test real samples (serum from two patients treated with chemo-immunotherapy) to evaluate MAVS utility as a predictive biomarker for therapeutic outcome. Fig. 5 shows an example of the SPR spectra obtained from these tests performed with samples of NR patient #1 and BR patient #1 at different dilution ratios (Fig. 5a and Fig. 5b, respectively), as described in Section 2.9. In particular, as shown in Fig. 5, in both cases, there is a response from the biosensor, indicating an interaction between the bioreceptor and the target analyte. As regards the NR patient #1, a significant signal is obtained from the 1:1000 diluted sample up to the 1:100 diluted sample (see Fig. 5a). On the other hand, the BR patient #1 registers a significant signal from the 1:10,000 diluted sample up to the 1:1000 diluted sample (Fig. 5b), indicating a 10-fold higher concentration of MAVS compared to the NR patient. Moreover, supplemental Figure S3 illustrates NR patient’s cohort (Cases #2, #3, #4, #5), showing the spectral response at different dilution factors. Similarly, the comprehensive analysis for the BR cohort is detailed in supplemental Figure S4, which displays the spectral shifts for BR patients #2, #3, #4, and #5 at various dilution factors.

A summary bar chart, shown in Figure S5 of the supplementary materials, reports the MAVS biosensor results obtained with the positive sample and two real samples (i.e., NR patient #1 and BR patient #1) highlighting how the signals obtained for NR patients are significant only at dilution factors 10 times lower.

The experimental data, i.e., the resonance wavelength shifts (Δλ) achieved at different dilution factors of the real samples, were used to estimate the MAVS concentration, as summarized for each sample in Tables S1-S10 (Supplementary materials file). In particular, each samples that showed a significant response respect to noise and that were far from saturation (samples diluted 1:1000, 1:500 and 1:250 of NR patient and 1:10,000, 1:5000 and 1:2500 of BR patient) can be used to estimate the corresponding MAVS concentration on the dose-response curve in diluted human serum (Fig. 2d), as reported for example in Figure S6 and Figure S7 of the supplementary materials file. More specifically, the MAVS concentration in real samples can be estimated by multiplying the predicted concentration (which is the concentration obtained from the Δλ evaluation) by the related dilution factor, as reported in Tables S1-S10 of the supplementary materials file. Notably, the fact that different dilution factors yield consistent estimates of the MAVS concentration in the original samples indicates the internal robustness of the quantification approach. This convergence of concentration values obtained from multiple points along the dose–response curve further supports the adequacy of the Langmuir model in accurately describing the analyte–receptor interaction. Therefore, the standard deviation obtained in the estimation of the MAVS concentration represents both model error and measurement error. In this frame, Table S11 shows the estimated MAVS concentration values for the NR and BR patients. More specifically, each MAVS concentration value is computed as the means of the values achieved at three different dilutions ratios.

**Fig. 6:**
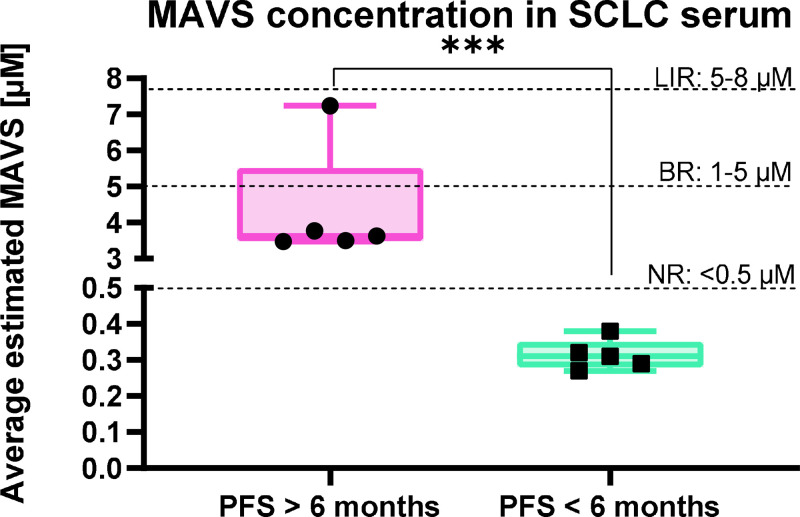
Average estimated MAVS concentration in real samples. SCLC patients’ sera were collected from patients with PFS > 6 months (*n* = 5) and PFS < 6 months (*n* = 5). BR: Best Responders. LIR: Long Immuno Responders. NR: Non Responders.

As shown in Fig. 6 and Table S11, patients with a Progression-Free Survival (PFS) > 6 months (comprising BR and Long Immuno-Responder [LIR] patients) exhibited significantly higher MAVS concentrations compared to those with a PFS < 6 months (NR patients). Specifically, the LIR patient showed the highest MAVS levels, ranging between 5 and 8 μM, while BR patients fell within the 1–5 μM range. Conversely, all NR patients consistently displayed MAVS concentrations below 0.5 μM. This clear stratification suggests that elevated circulating MAVS levels are strongly correlated with favourable therapeutic outcomes and prolonged PFS in SCLC patients treated with chemo-immunotherapy.

## Discussion

SCLC is a highly aggressive tumor type with recent improvements in clinical outcome based on immunotherapy addition in a subgroup of patients (about 15 % of cases); however, without the possibility of identifying the subgroup of immune responsive patients with clinical testing of biomarkers [19]. Current biomarker strategies often rely on tumor tissue analysis, which in SCLC is frequently limited by small biopsy samples and high tumor heterogeneity, complicating comprehensive molecular profiling. Moreover, conventional tissue-based biomarkers such as PD-L1 expression and tumor mutation burden have shown limited predictive value in SCLC [20].

In this context, liquid biopsy has emerged as a minimally invasive and repeatable alternative to tissue biopsy, offering real-time insights into tumor dynamics through analysis of circulating tumor cells, DNA, and immune-related biomarkers in peripheral blood. Liquid biopsy not only facilitates early detection and monitoring of treatment response but also helps in overcoming spatial and temporal tumor heterogeneity [[21], [22], [23]]. Within the immune landscape of SCLC, there is growing interest in the role of innate immune sensors that mediate antiviral and antitumor immunity [24,25]. One such sensor is the MAVS, which acts as a key adaptor in the RNA virus-sensing pathway. MAVS is located on the outer mitochondrial membrane and is critical for initiating type I interferon responses after viral recognition by cytosolic RNA receptors [26]. The clinical significance of MAVS in SCLC pathogenesis lies in its ability to bridge innate sensing with adaptive immune responses. Beyond its antiviral function, MAVS modulates immune cell activation and inflammation, influencing macrophage polarization and T cell responses [27]. Importantly, MAVS forms functional complexes with STING, amplifying innate immune signaling pathways upon DNA-damaging therapies, thereby inducing tumor immune responses [28]. In the context of disease progression, sustained MAVS activation may prevent immune evasion by maintaining an "inflamed" tumor microenvironment, whereas its downregulation could facilitate rapid resistance and metastasis, characteristic of the SCLC-ES phenotype.

Our group and others have demonstrated that the activation of immune response in SCLC in preclinical models and in PBMCs from SCLC patients under immunotherapy is associated with sustained activation of innate immune sensor pathways like STING and MAVS, that are strongly increased in presence of intrinsic DNA damage features (germline mutations in DDR associated genes in all lung cancer types) and under pressure of chemo-, radio- and other targeted DDR inhibitors [4,28]. Being in the clinical scenario the approach to SCLC therapy is still based on one-size-fit-all indications without selection by biomarkers, differently from NSCLC, it is critical to find any applicable biomarker to predict and monitor immune response in SCLC patients to anticipate and guide clinical decisions.

Based on our preclinical data and study on PBMCs, we designed a proof-of-concept study on SCLC patients (*n* = 8 patients with a median age of 63 years) treated in our Institution to measure circulating MAVS expression levels in blood samples collected during chemo-immunotherapy treatment. To this end, we applied a novel biosensor that has been already validated in other biological settings. This is to our knowledge the first study applying this technique to an oncology scenario, with special attention to the SCLC population that is characterized by a fast onset of treatment resistance and few possibilities of clinical biomarkers monitoring out of clinical trials. Specifically, to obtain the MAVS biosensor chip, the conventional SPR-POF probe was functionalized with a specific antibody for the target protein. The proposed MAVS biosensor was tested with different MAVS concentrations, diluted in the phosphate buffer and in the real matrix of healthy patient serum diluted 1:500, achieving LODs of around 0.17 and 0.13 nM, respectively. Then, selectivity tests were performed in the presence of potential interfering proteins (commonly found in human serum), showing non-relevant responses. Finally, the MAVS biosensor was tested in a real matrix, i.e., the serum of SCLC patients exhibiting different responses to chemo-immunotherapy. The experimental data confirmed a clear stratification of the cohort based on MAVS levels. As shown in Fig. 6, patients with a PFS > 6 months, including BR and notably the Long Immuno-Responder LIR patient, exhibited significantly higher MAVS concentrations compared to those with a PFS < 6 months. While NR patients consistently displayed MAVS concentrations below 0.5 μM, the BR group fell within the 1–5 μM range, and the LIR patient showed the highest expression levels, reaching 5–8 μM.

In this study, we correlated clinical response with MAVS levels as detected by biosensors and we identified a good predictive association. In fact, we demonstrated the feasibility of MAVS biosensors application to BR and NR SCLC, with the detection of 10-fold higher concentration of MAVS in BR patients compared to the NR patients. This result aligns with one of our preclinical data, as shown by WB, demonstrating a feasible and fast application of this testing to reproduce preclinical and translational findings. Being aware of limitations represented by low numbers of patients, we propose this proof-of-concept study as the first approach to be further validated prospectively. Moreover, while the sensor effectively predicts BR patients, the analysis exhibits higher variability when distinguishing between BR and LIR levels. Furthermore, a significant limitation is the lack of cross-validation using alternative methods or biological tests to verify the sensor's accuracy in quantifying circulating MAVS protein.

Indeed, the proposed sensor configuration was designed to ensure cost-effectiveness, portability, and operational simplicity, characteristics that make it particularly suitable for POCT applications. The fabrication of the SPR-POF platforms involves an estimated unit cost of approximately 5 USD, which includes materials, machining, and processing [15]. This low-cost system gives high feasibility due to the intrinsic simplicity, portability and rapid response, which make it well-suited for development outside a conventional laboratory setting with high selectivity and sensitivity. This sensor configuration not only ensures accessibility and enables scalable implementation for large-scale screening and routine monitoring.

## Conclusion

In this work, a POCT based on a SPR-POF sensor combined with a specific antibody for the detection of MAVS was developed and tested in both PBS and diluted human serum. Specifically, the proposed biosensor allows for the rapid measurement of MAVS protein with high sensitivity, operating at the nanomolar level, and with good selectivity. Moreover, as a proof-of-concept, the present study identifies for the first time the feasibility of applying the MAVS biosensor to a clinical cohort of SCLC under immunotherapy, with good association with clinical response.

## Funding

This work was supported by 10.13039/100020581Fondazione AIRC (MFAG Project Number: 26237 to C.M.D.C

## CRediT authorship contribution statement

**Luisa Amato:** Writing – original draft, Visualization, Validation, Methodology, Investigation, Formal analysis, Data curation, Conceptualization. **Ines Tavoletta:** Writing – original draft, Visualization, Validation, Software, Methodology, Investigation, Formal analysis, Data curation, Conceptualization. **Caterina De Rosa:** Writing – original draft, Visualization, Methodology, Investigation, Formal analysis, Data curation. **Mimimorena Seggio:** Writing – original draft, Formal analysis, Data curation. **Francesco Arcadio:** Software, Methodology, Investigation. **Sara Capaldo:** Methodology, Investigation. **Faiz Ul Haq:** Validation, Methodology. **Concetta Tuccillo:** Resources, Data curation. **Carla Esposito:** Visualization, Resources. **Gaetano di Guida:** Software, Resources, Investigation. **Francesca Iommelli:** Writing – review & editing, Visualization, Validation, Supervision, Project administration, Formal analysis, Data curation. **Viviana De Rosa:** Writing – review & editing, Supervision, Project administration, Formal analysis, Data curation. **Alfonso Reginelli:** Writing – review & editing, Validation, Supervision, Resources. **Salvatore Cappabianca:** Writing – review & editing, Supervision. **Floriana Morgillo:** Supervision, Project administration. **Fortunato Ciardiello:** Supervision, Resources, Project administration. **Valerio Nardone:** Supervision, Resources, Methodology, Formal analysis. **Nunzio Cennamo:** Writing – review & editing, Validation, Supervision, Resources, Methodology, Investigation, Conceptualization. **Carminia Maria Della Corte:** Writing – review & editing, Writing – original draft, Validation, Supervision, Resources, Project administration, Funding acquisition, Conceptualization. **Luigi Zeni:** Writing – review & editing, Writing – original draft, Supervision, Resources, Project administration, Methodology, Conceptualization.

## Declaration of competing interest

Dr. Ciardiello F. Receipt of honoraria or consultation fees for speaker, consultancy, or advisory roles: Amgen, Merck KGaA, MSD, Pierre Fabre, Pfizer, Roche, Servier; Institutional financial interests, financial support for clinical trials or contracted research; Amgen, Merck KGaA, MSD, Pierre Fabre, Pfizer, Roche, Servier.

Dr. Morgillo F. Received honoraria or consultation fees for speaker, consultancy, or advisory roles: Roche, Servier, Incyte, ESMO, and MSD.

Dr. Nardone V. Received honorary fees from AstraZeneca, Gilead and SunPharma.

Dr. Della Corte C.M. reported receiving honoraria or consultation fees for speaker, consultancy, or advisory roles from AstraZeneca, Pfizer, Regeneron, Roche, Genetic, Merck, Novartis, BMS, MSD, Amgen, Takeda and travel grants from Daiichi Sankyo, Amgen, MSD, Novartis, AstraZeneca outside the submitted work.

The remaining authors declare no conflict of interest.

## Appendix Supplementary materials

Image, application 1
