# Supplementary material for: Proof-of-concept study of clinical use of blood-based MAVS biosensor in predicting immunotherapy response in SCLC patients
Source: Transl Oncol. 2026 Apr 4;67:102756. doi: 10.1016/j.tranon.2026.102756 (PMC13090305; doi:10.1016/j.tranon.2026.102756)
Supplement: Supplementary file 1 [file mmc1.docx]

**Supplementary materials**

**Article Title: Proof-of-concept study of clinical use of blood-based MAVS biosensor in predicting immunotherapy response in SCLC patients**

**Authors:** Luisa Amato^†1^, Ines Tavoletta^†2^, Caterina De Rosa^1^, Mimimorena Seggio^3^, Francesco Arcadio^2^, Sara Capaldo^1^, Faiz Ul Haq^1^, Concetta Tuccillo^1^, Carla Esposito^1^, Gaetano di Guida^1^, Francesca Iommelli^4^, Viviana De Rosa^4^, Alfonso Reginelli^5^, Salvatore Cappabianca^5^, Floriana Morgillo^1^, Fortunato Ciardiello^1^, Valerio Nardone^5^, Nunzio Cennamo^‡2^, Carminia Maria Della Corte^‡^*^1^ and Luigi Zeni^‡^*^2^

**Affiliations:**

^1^Department of Precision Medicine, University of Campania Luigi Vanvitelli, 80131 Naples, Italy; [luisa.amato@unicampania.it](mailto:luisa.amato@unicampania.it); [caterina.derosa1@unicampania.it](mailto:caterina.derosa1@unicampania.it); [saracapaldo99@gmail.com](mailto:saracapaldo99@gmail.com); [faizul.haq@unicampania.it](mailto:faizul.haq@unicampania.it); [concetta.tuccillo@unicampania.it](mailto:concetta.tuccillo@unicampania.it); [carla.esposito1201@gmail.com](mailto:carla.esposito1201@gmail.com); [gaetano.diguida1997@gmail.com](mailto:gaetano.diguida1997@gmail.com); [floriana.morgillo@unicampania.it](mailto:floriana.morgillo@unicampania.it); [fortunato.ciardiello@unicampania.it](mailto:fortunato.ciardiello@unicampania.it); [carminiamaria.dellacorte@unicampania.it](mailto:carminiamaria.dellacorte@unicampania.it);

^2^Department of Engineering, University of Campania Luigi Vanvitelli, 81031 Aversa, Italy; ines.tavoletta@unicampania.it; francesco.arcadio@unicampania.it; nunzio.cennamo@unicampania.it; luigi.zeni@unicampania.it

^3^Department of Engineering, Telematic University Pegaso, 80132 Naples, Italy; [mimimorena.seggio@unipegaso.it](mailto:mimimorena.seggio@unipegaso.it)

^4^Institute of Biostructures and Bioimaging, National Research Council, 80145 Naples, Italy; [francesca.iommelli@ibb.cnr.it](mailto:francesca.iommelli@ibb.cnr.it); [viviana.derosa@ibb.cnr.it](mailto:viviana.derosa@ibb.cnr.it);

^5^Radiology and Radiotherapy, Department of Precision Medicine, University of Campania Luigi Vanvitelli, 80138 Naples, Italy; [alfonso.reginelli@unicampania.it](mailto:alfonso.reginelli@unicampania.it); [salvatore.cappabianca@unicampania.it](mailto:salvatore.cappabianca@unicampania.it); [valerio.nardone@unicampania.it](mailto:valerio.nardone@unicampania.it);

^†^These authors contributed equally to this work as co-first authors.

^‡^These authors contributed equally to this work as co-last authors.

***Co-corresponding authors**: Carminia Maria Della Corte, Dept. of Precision Medicine, University of Campania “L. Vanvitelli”, Via S. Pansini 5, 80131 Napoli, Italy, carminiamaria.dellacorte@unicampania.it; and Luigi Zeni, Dept. of Engineering, University of Campania “L. Vanvitelli”, Via Roma 29, 81031 Aversa, Italy, luigi.zeni@unicampania.it.

**
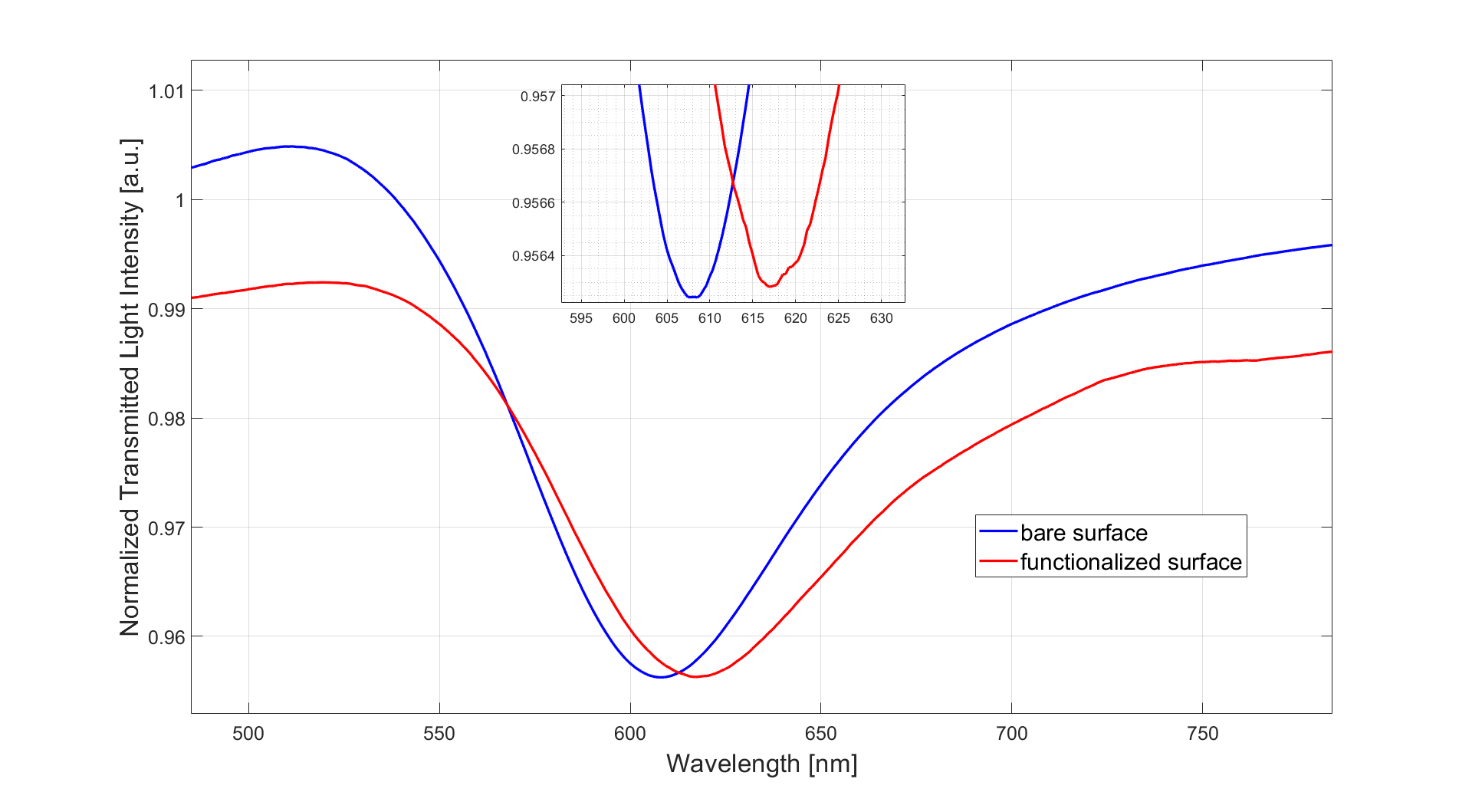
**

**Figure S1:** SPR spectra attained by using PBS as bulk solution before (blue line) and after (red line) the functionalization process (via α-lipoic acid, NHS/EDC mixture, antibody, and ethanolamine).


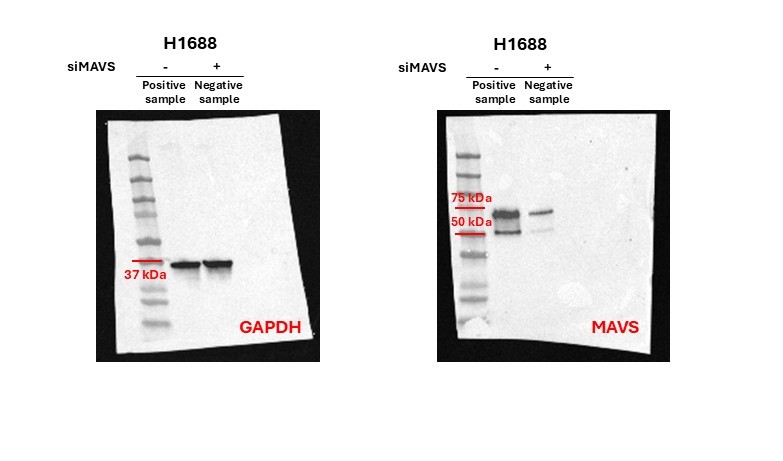


**Figure S2:** Original uncropped western blot images, related to Figure 4b.


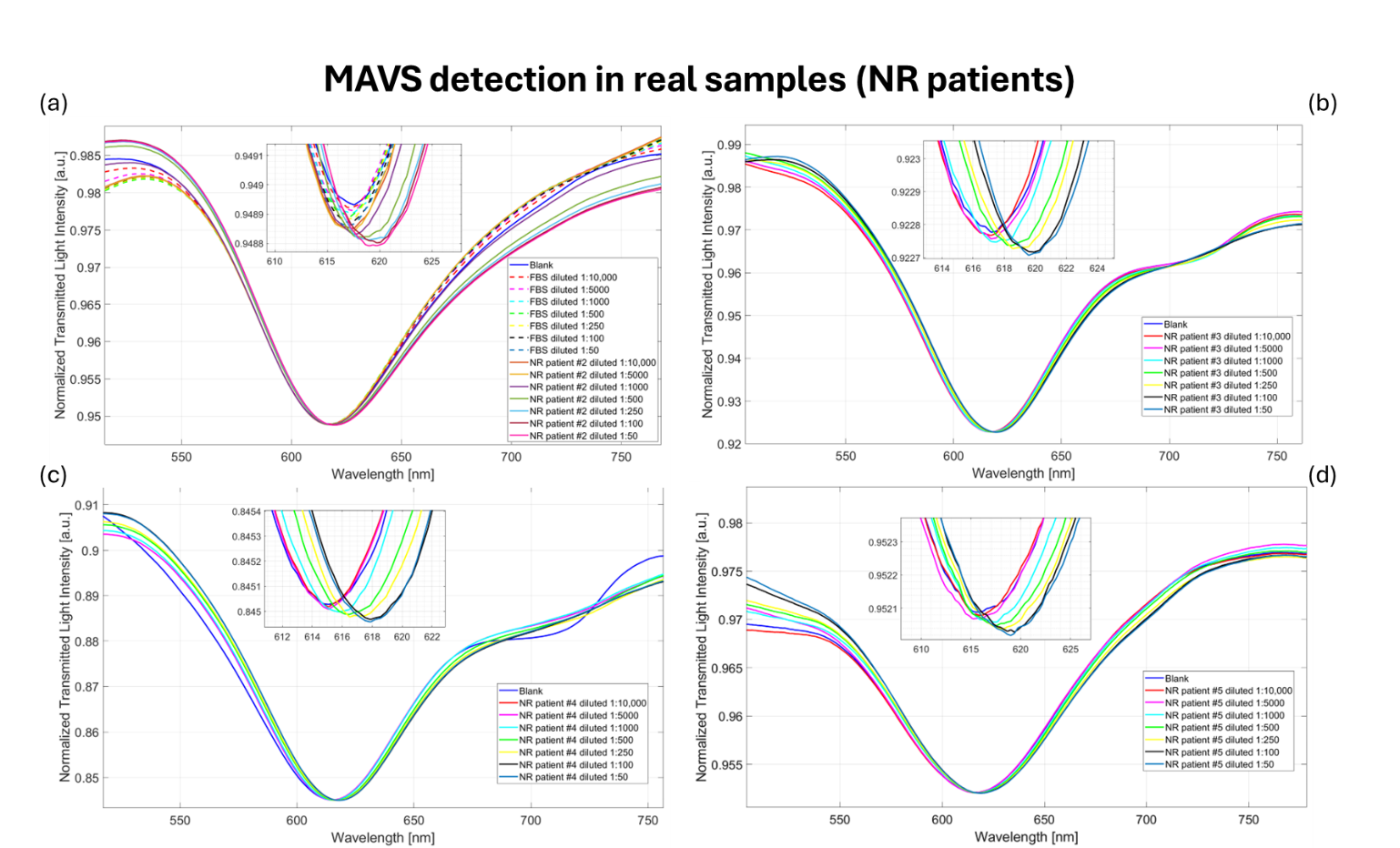


**Figure S3**: SPR spectra recorded for the Non-Responder (NR) cohort. Panels (a) through (d) show the normalized transmitted light intensity and resonance shifts for NR patients #2, #3, #4, and #5 at dilution factors ranging from 1:10,000 to 1:50. Panel (a) illustrates the comparison between fetal bovine serum (FBS) and NR patient #2 spectra at different dilution factors, confirming the biosensor's ability to distinguish clinical target concentrations from the complex biological background.


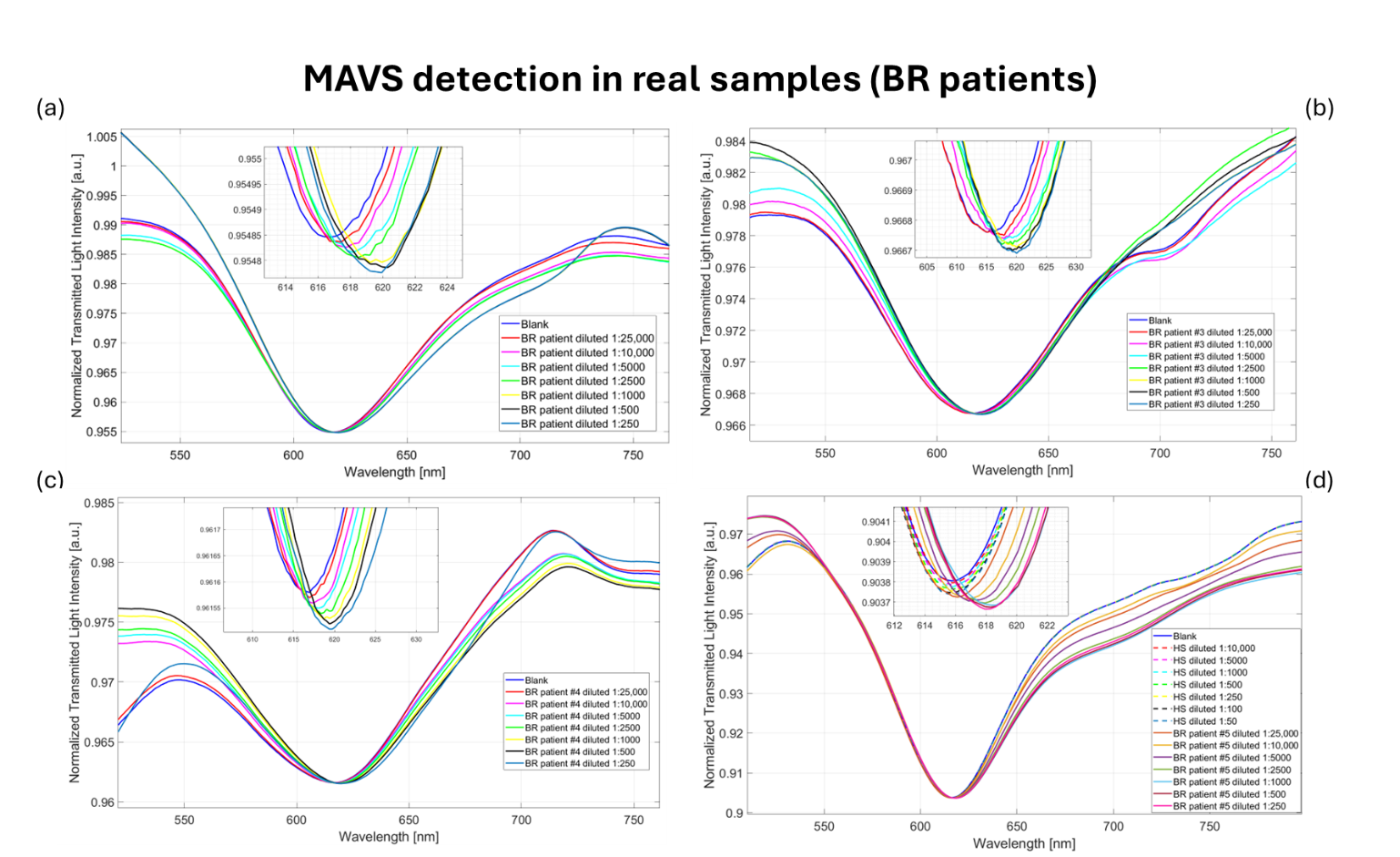


**Figure S4**: SPR spectra obtained for Best Responder (BR) patients at varied dilution ratios (from 1:25,000 to 1:250). Panels (a) through (d) represent plasmonic spectra for individual BR patients, showing significant wavelength variation even at high dilution factors. Panel (d) illustrates the comparison between human serum (HS) controls and BR patient #5 spectra at different dilution factors, confirming the biosensor's ability to distinguish clinical target concentrations from the complex biological background.


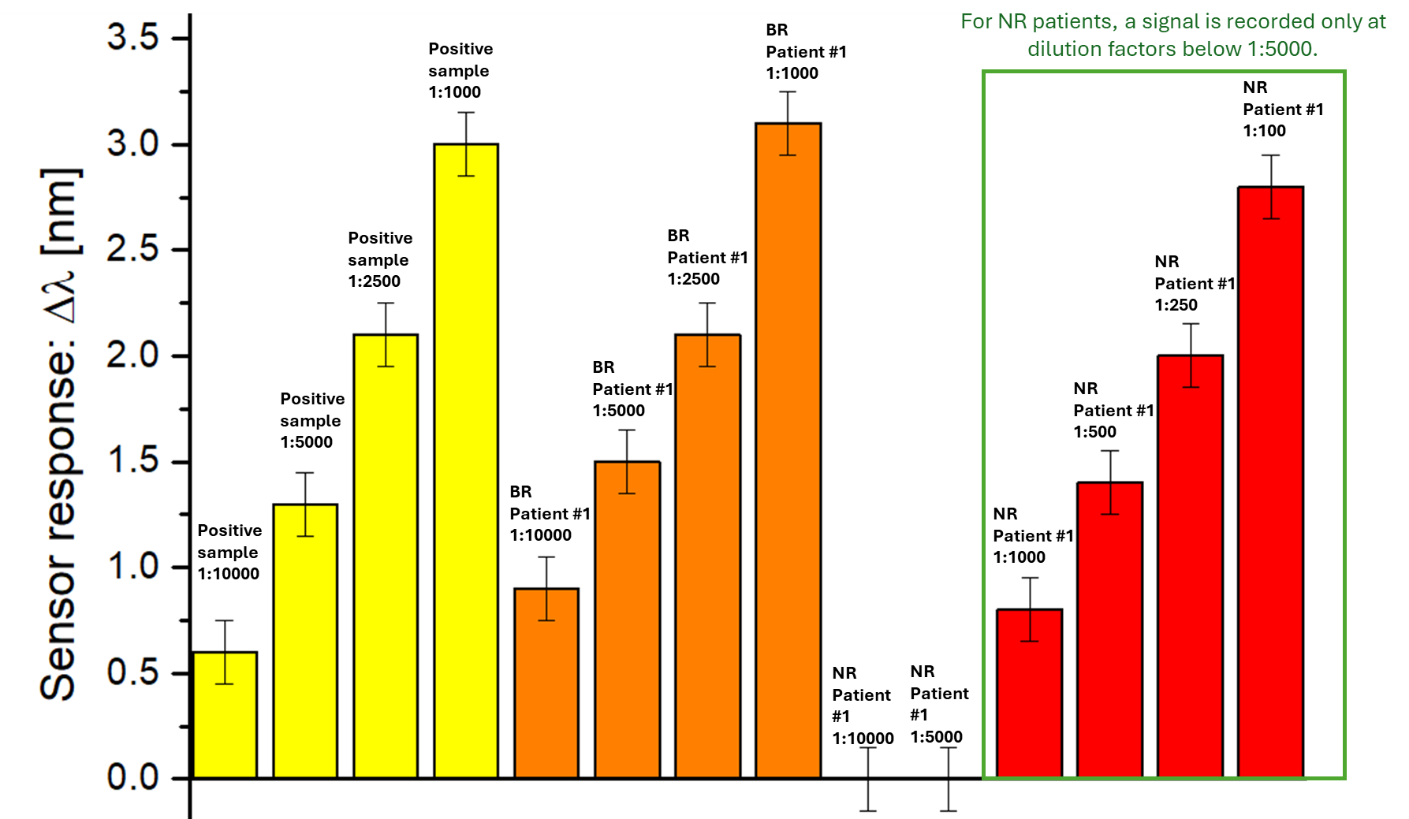


**Figure S5:** Summary bar chart showing the sensor response (∆λ, in nm) of the proposed optical sensor for the MAVS detection in different diluted samples of the positive control (yellow bar), BR patient #1 (orange bar) and NR patient #1 (red bar).


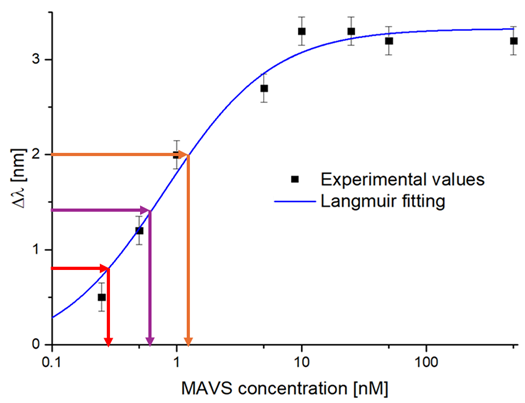


**Figure S6:** Graphical representation of the MAVS concentration estimation in NR patient #1 from the dose-response curve reported in Figure 2d.

**Table S1:** MAVS biosensor response relative to the NR patient #1 tested at different dilution factors and estimation of the MAVS concentration from the binding dose-response curve reported in Figure 2d.

| **Sample** | **Δλ [nm]** | **Predicted MAVS concentration** | **Estimated MAVS concentration** |
| --- | --- | --- | --- |
| NR patient #1 1:10,000 | 0 | Near LOD value | / |
| NR patient #1 1:5000 | 0 | Near LOD value | / |
| NR patient #1 1:1000 | 0.8 | 0.285 nM | 0.285 nM*1000=0.29 μM |
| NR patient #1 1:500 | 1.4 | 0.62 nM | 0.62 nM*500=0.31 μM |
| NR patient #1 1:250 | 2 | 1.26 nM | 1.26 nM*250=0.32 μM |
| NR patient #1 1:100 | 2.8 | Near saturation value | / |
| NR patient #1 1:50 | 3 | Near saturation value | / |


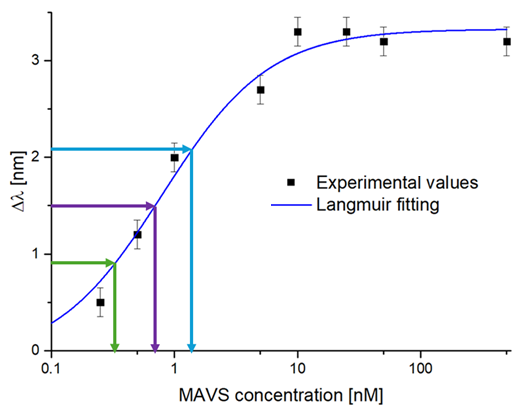


**Figure S7:** Graphical representation of the MAVS concentration estimation in BR patient #1 from the dose-response curve in human diluted serum (Figure 2d).

**Table S2:** MAVS biosensor response relative to the BR patient #1 tested at different dilution factors and estimation of the MAVS concentration from the binding dose-response curve reported in Figure 2d.

| **Sample** | **Δλ [nm]** | **Predicted MAVS concentration** | **Estimated MAVS concentration** |
| --- | --- | --- | --- |
| BR patient #1 1:25,000 | 0.3 | Near LOD value | / |
| BR patient #1 1:10,000 | 0.9 | 0.33 nM | 0.33 nM*10,000=3.3 μM |
| BR patient #1 1:5000 | 1.5 | 0.7 nM | 0.7 nM*5000= 3.5 μM |
| BR patient #1 1:2500 | 2.1 | 1.43 nM | 1.43 nM*2500=3.6 μM |
| BR patient #1 1:1000 | 3.1 | Near saturation value | / |
| BR patient #1 1:500 | 3.1 | Near saturation value | / |
| BR patient #1 1:250 | 3.1 | Near saturation value | / |

**Table S3:** MAVS biosensor response relative to the NR patient #2 tested at different dilution factors and estimation of the MAVS concentration from the binding dose-response curve reported in Figure 2d.

| **Sample** | **Δλ [nm]** | **Predicted MAVS concentration** | **Estimated MAVS concentration** |
| --- | --- | --- | --- |
| NR patient #2 1:10,000 | 0.1 | Near LOD value | / |
| NR patient #2 1:5000 | 0.2 | Near LOD value | / |
| NR patient #2 1:1000 | 0.9 | 0.33 nM | 0.33 nM*1000=0.33 μM |
| NR patient #2 1:500 | 1.6 | 0.785 nM | 0.785 nM*500=0.39 μM |
| NR patient #2 1:250 | 2.2 | 1.625 nM | 1.625 nM*250=0.41 μM |
| NR patient #2 1:100 | 2.9 | Near saturation value | / |
| NR patient #2 1:50 | 2.9 | Near saturation value | / |

**Table S4:** MAVS biosensor response relative to the NR patient #3 tested at different dilution factors and estimation of the MAVS concentration from the binding dose-response curve reported in Figure 2d.

| **Sample** | | **Δλ [nm]** | **Predicted MAVS concentration** | | | **Estimated MAVS concentration** |
| --- | --- | --- | --- | --- | --- | --- |
| NR patient #3 1:10,000 | | -0.2 | Under LOD value | | | / |
| NR patient #3 1:5000 | | 0 | Under LOD value | | | / |
| NR patient #3 1:1000 | 0.7 | | | 0.25 nM | 0.25 nM*1000=0.25 μM | |
| NR patient #3 1:500 | | 1.4 | 0.62 nM | | | 0.62 nM*500=0.31 μM |
| NR patient #3 1:250 | | 1.8 | 0.995 nM | | | 0.995 nM*250=0.25 μM |
| NR patient #3 1:100 | | 2.7 | Near saturation value | | | / |
| NR patient #3 1:50 | | 2.9 | Near saturation value | | | / |

**Table S5:** MAVS biosensor response relative to the NR patient #4 tested at different dilution factors and estimation of the MAVS concentration from the binding dose-response curve reported in Figure 2d.

| **Sample** | **Δλ [nm]** | **Predicted MAVS concentration** | | **Estimated MAVS concentration** |
| --- | --- | --- | --- | --- |
| NR patient #4 1:10,000 | 0 | Under LOD value | | / |
| NR patient #4 1:5000 | 0 | Under LOD value | | / |
| NR patient #4 1:1000 | 0.8 | 0.285 nM | 0.285 nM*1000=0.28 μM | |
| NR patient #4 1:500 | 1.4 | 0.62 nM | | 0.62 nM*500=0.31 μM |
| NR patient #4 1:250 | 1.9 | 1.12 nM | | 1.12 nM*250=0.28 μM |
| NR patient #4 1:100 | 2.9 | Near saturation value | | / |
| NR patient #4 1:50 | 2.8 | Near saturation value | | / |

**Table S6:** MAVS biosensor response relative to the NR patient #5 tested at different dilution factors and estimation of the MAVS concentration from the binding dose-response curve reported in Figure 2d.

| **Sample** | **Δλ [nm]** | **Predicted MAVS concentration** | | **Estimated MAVS concentration** |
| --- | --- | --- | --- | --- |
| NR patient #5 1:10,000 | 0 | Under LOD value | | / |
| NR patient #5 1:5000 | -0.1 | Under LOD value | | / |
| NR patient #5 1:1000 | 0.8 | 0.285 nM | 0.285 nM*1000=0.28 μM | |
| NR patient #5 1:500 | 1.5 | 0.7 nM | | 0.7 nM*500=0.35 μM |
| NR patient #5 1:250 | 2 | 1.26 nM | | 1.26 nM*250=0.32 μM |
| NR patient #5 1:100 | 2.8 | Near saturation value | | / |
| NR patient #5 1:50 | 3 | Near saturation value | | / |

**Table S7:** MAVS biosensor response relative to the BR patient #2 tested at different dilution factors and estimation of the MAVS concentration from the binding dose-response curve reported in Figure 2d.

| **Sample** | **Δλ [nm]** | **Predicted MAVS concentration** | **Estimated MAVS concentration** |
| --- | --- | --- | --- |
| BR patient #2 1:25,000 | 0.4 | Near LOD value | / |
| BR patient #2 1:10,000 | 1 | 0.38 nM | 0.38 nM*10,000=3.8 μM |
| BR patient #2 1:5000 | 1.6 | 0.785 nM | 0.785 nM*5000= 3.9 μM |
| BR patient #2 1:2500 | 2.1 | 1.43 nM | 1.43 nM*2500=3.6 μM |
| BR patient #2 1:1000 | 3 | Near saturation value | / |
| BR patient #2 1:500 | 3 | Near saturation value | / |
| BR patient #2 1:250 | 2.9 | Near saturation value | / |

**Table S8:** MAVS biosensor response relative to the BR patient #3 (LIR) tested at different dilution factors and estimation of the MAVS concentration from the binding dose-response curve reported in Figure 2d.

| **Sample** | **Δλ [nm]** | **Predicted MAVS concentration** | **Estimated MAVS concentration** |
| --- | --- | --- | --- |
| BR patient #3 (LIR) 1:25,000 | 0.8 | 0.285 nM | 0.285 nM *25,000=7.13 μM |
| BR patient #3 (LIR) 1:10,000 | 1.5 | 0.7 nM | 0.7 nM *10,000= 7 μM |
| BR patient #3 (LIR) 1:5000 | 2.4 | 1.52 nM | 2.52 nM*5000= 7.6 μM |
| BR patient #3 (LIR) 1:2500 | 2.9 | Near saturation value | / |
| BR patient #3 (LIR) 1:1000 | 3.5 | Near saturation value | / |
| BR patient #3 (LIR) 1:500 | 3.5 | Near saturation value | / |
| BR patient #3 (LIR) 1:250 | 3.5 | Near saturation value | / |

**Table S9:** MAVS biosensor response relative to the BR patient #4 tested at different dilution factors and estimation of the MAVS concentration from the binding dose-response curve reported in Figure 2d.

| **Sample** | **Δλ [nm]** | **Predicted MAVS concentration** | **Estimated MAVS concentration** |
| --- | --- | --- | --- |
| BR patient #4 1:25,000 | 0.4 | Near LOD value | / |
| BR patient #4 1:10,000 | 1 | 0.38 nM | 0.38 nM*10,000=3.8 μM |
| BR patient #4 1:5000 | 1.5 | 0.7 nM | 0.7 nM*5000= 3.5 μM |
| BR patient #4 1:2500 | 2 | 1.26 nM | 1.26 nM*2500=3.2 μM |
| BR patient #4 1:1000 | 2.6 | Near saturation value | / |
| BR patient #4 1:500 | 3.2 | Near saturation value | / |
| BR patient #4 1:250 | 3.3 | Near saturation value | / |

**Table S10:** MAVS biosensor response relative to the BR patient #5 tested at different dilution factors and estimation of the MAVS concentration from the binding dose-response curve reported in Figure 2d.

| **Sample** | **Δλ [nm]** | **Predicted MAVS concentration** | **Estimated MAVS concentration** |
| --- | --- | --- | --- |
| BR patient #5 1:25,000 | 0.4 | Near LOD value | / |
| BR patient #5 1:10,000 | 1 | 0.38 nM | 0.38 nM*10,000=3.8 μM |
| BR patient #5 1:5000 | 1.5 | 0.7 nM | 0.7 nM*5000= 3.5 μM |
| BR patient #5 1:2500 | 2.1 | 1.43 nM | 1.43 nM*2500=3.6 μM |
| BR patient #5 1:1000 | 2.7 | Near saturation value | / |
| BR patient #5 1:500 | 2.7 | Near saturation value | / |
| BR patient #5 1:250 | 2.7 | Near saturation value | / |

**Table S11:** MAVS detection in real samples**.** MAVS biosensor response relative to NR and BR patients tested at different dilution factors and estimation of the MAVS concentration from the binding dose-response curve reported Figure 2d.

| Sample | Estimated MAVS concentration via SPR-POF biosensor [μM] |
| --- | --- |
| NR patient #1 | 0.31±0.02 |
| NR patient #2 | 0.38±0.04 |
| NR patient #3 | 0.27 ±0.03 |
| NR patient #4 | 0.29±0.02 |
| NR patient #5 | 0.32±0.04 |
| BR patient #1 | 3.47±0.15 |
| BR patient #2 | 3.77±0.15 |
| BR patient #3 (LIR) | 7.24±0.32 |
| BR patient #4 | 3.50±0.30 |
| BR patient #5 | 3.63±0.15 |
